# Brain–Computer Interface for EEG-Based Authentication: Advancements and Practical Implications

**DOI:** 10.3390/s25164946

**Published:** 2025-08-10

**Authors:** Lamia Alahaideb, Abeer Al-Nafjan, Hessah Aljumah, Mashael Aldayel

**Affiliations:** 1 Computer Science Department, College of Computer and Information Sciences, Imam Mohammad Ibn Saud Islamic University, Riyadh 11432, Saudi Arabia; 2 Information Technology Department, College of Computer and Information Sciences, King Saud University, Riyadh 11543, Saudi Arabia

**Keywords:** electroencephalography (EEG), brain–computer interface (BCI), authentication, event-related potentials (ERP), convolutional neural networks (CNN)

## Abstract

Authentication is a critical component of digital security, and traditional methods often encounter significant vulnerabilities and limitations. This study addresses the emerging field of EEG-based authentication systems, highlighting their theoretical advancements and practical applicability. We conducted a systematic review of the existing literature, followed by an experimental evaluation to assess the feasibility, limitations, and scalability of these systems in real-world scenarios. Data were collected from nine subjects using various approaches. Our results indicate that the CNN model achieved the highest accuracy of 99%, while Random Forest (RF) and Gradient Boosting (GB) classifiers also demonstrated strong performance with 94% and 93%, respectively. In contrast, classifiers such as Support Vector Machine (SVM) and K-Nearest Neighbors (KNN) displayed significantly lower effectiveness, underscoring their limitations in capturing the complexities of EEG data. The findings suggest that EEG-based authentication systems have significant potential to enhance security measures, offering a promising alternative to traditional methods and paving the way for more robust and user-friendly authentication solutions.

## 1. Introduction

Authentication plays a crucial role in safeguarding digital infrastructure and personal information against unauthorized access. As the first line of defense in information security systems, authentication mechanisms are essential for ensuring the integrity, confidentiality, and availability of sensitive data. However, conventional methods such as passwords, personal identification numbers (PINs), and physical tokens are increasingly recognized as insufficient. These approaches are frequently subject to security vulnerabilities such as brute-force attacks, phishing, and social engineering, while also imposing a cognitive burden on users due to the need to recall complex or frequently changing credentials [1].

Recent technological advancements have brought Brain–Computer Interfaces (BCIs) to the forefront of research and innovation, offering new possibilities for interaction between humans and machines. Non-invasive electroencephalography (EEG)-based BCIs have garnered particular attention due to their portability, wireless capabilities, and relative ease of use. EEG technology, which captures electrical brain activity via scalp electrodes, has found broad applicability in fields such as neurorehabilitation, cognitive assessment, gaming, and assistive technologies. Research in the BCI field has increased since then, and BCIs are now used in numerous areas, such as education, entertainment, and, recently, authentication [2]. Within this context, EEG signals have emerged as a novel and promising modality for biometric authentication. EEG signals reflect intrinsic and covert neurophysiological processes that are unique to each individual. Their high inter-subject variability and resistance to external replication make EEG-based biometrics exceptionally difficult to forge, thereby offering enhanced security. Furthermore, the dynamic nature of brain activity provides an additional dimension for liveness detection, making EEG particularly suitable for robust and continuous authentication systems [3].

While traditional biometric modalities—such as fingerprints and facial recognition—are widely deployed, they present fundamental limitations. These traits, once compromised, are irrevocable and cannot be reissued, raising critical concerns in security-sensitive domains. EEG-based authentication, by contrast, offers revocability through task-dependent variability, enabling the regeneration of biometric templates using different cognitive stimuli. Moreover, EEG signals are internal, non-visible, and transient, making them inherently resistant to spoofing, replication, or covert acquisition [4,5]. The live nature of brain activity further supports continuous authentication, while the uniqueness and non-observability of EEG signals provide a privacy-preserving biometric layer [6]. These characteristics collectively underscore EEG’s added value as a secure and adaptive alternative to traditional biometrics.

Recent literature reflects a growing consensus that EEG is not only promising but also increasingly prioritized within the landscape of biometric authentication. For instance, Khan et al. [3] provide a comprehensive overview of EEG-based authentication systems, highlighting their evolution, signal processing strategies, and classification techniques. Their work illustrates how EEG has emerged as a viable solution to the limitations of traditional biometric modalities. Furthermore, Islam et al. [7] highlight the broader trend of expanding EEG applications, originally limited to clinical and assistive technologies, into areas such as user identification and security. Together, these findings reinforce the strategic relevance of EEG as a next-generation biometric modality.

A growing corpus of academic literature has explored the integration of EEG signals into biometric authentication frameworks, demonstrating both the feasibility and the technical challenges of this emerging approach. Prior studies have investigated diverse experimental paradigms, signal processing techniques, and classification models, establishing a strong empirical and theoretical foundation for further inquiry. This rich body of research attests to the increasing academic and practical interest in EEG-based authentication systems, while simultaneously highlighting key areas in need of refinement and standardization.

Against this backdrop, the present study seeks to systematically examine the current state of EEG-based authentication by reviewing the existing literature, identifying prevailing methodologies, and assessing the strengths and limitations of various approaches. In addition to this analytical component, the study aims to design and empirically evaluate a prototype authentication system that leverages EEG signals. Through this dual objective, the research contributes to the development of secure, user-friendly, and technologically advanced authentication solutions grounded in neurophysiological data.

This paper is organized as follows: Section 2 provides a systematic literature review. Section 3 presents the framework of the proposed EEG-based authentication system. Section 4 presents experiment design. Section 5 shows the results and discussion. Section 6 concludes the research.

## 2. Systematic Literature Review (SLR)

We review the existing literature on machine learning and deep learning approaches to biometric authentication, focusing specifically on EEG-based systems. By examining these techniques, we aim to elucidate state-of-the-art methods, their respective effectiveness, and the challenges they address in enhancing security and user experience in BCI-based authentication systems.

To systematically identify relevant contributions, an SLR was conducted, targeting peer-reviewed journal articles that address the themes of EEG, brain–computer interfaces, and authentication systems. The literature search was executed using the Web of Science (WoS) database, employing a refined set of keywords: “electroencephalography”, “brain–computer interface”, “authentication”, “identification”, “verification”, and “biometrics”. The review was restricted to English-language journal publications dated between 2018 and 2025.

The initial query yielded 1701 articles. Following the application of predefined inclusion criteria—namely, peer-reviewed status, English language, and journal publication type—non-relevant records such as conference proceedings, book chapters, editorials, and non-English works were excluded, resulting in 853 eligible articles. A first-level screening, based on titles, abstracts, keywords, and conclusions, eliminated studies that referenced EEG only tangentially, narrowing the selection to 312 articles.

**Figure 1:**
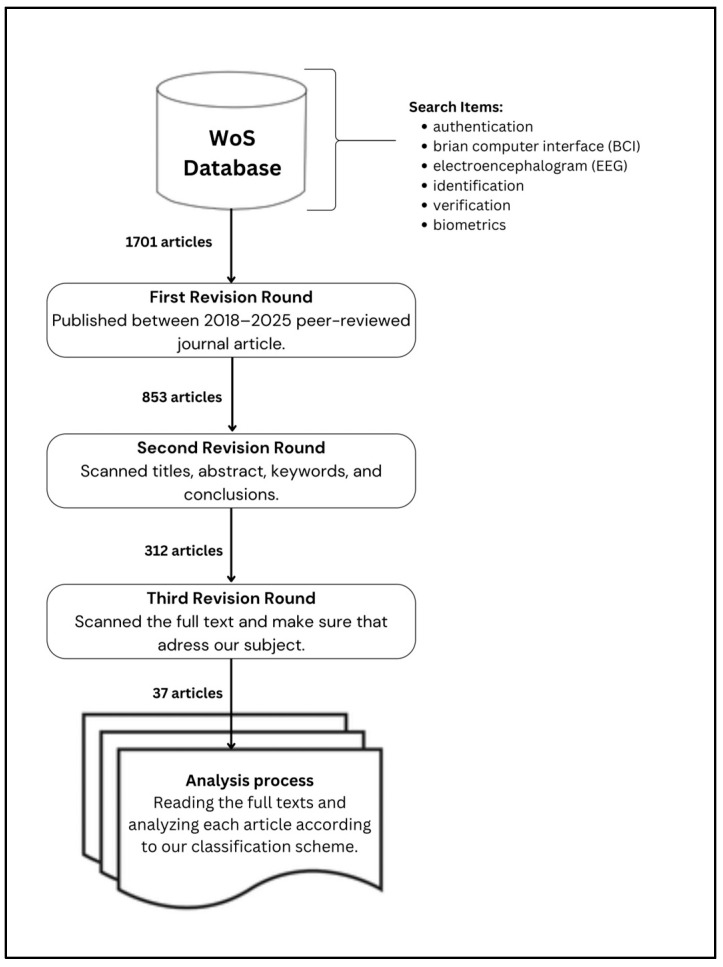
Research review protocol.

A second-level review entailed full-text examination to ensure conceptual and methodological alignment with the study’s aims, leading to the inclusion of 37 studies. These articles were then subjected to a third and final round of detailed qualitative analysis, categorized using a classification framework tailored to the research objectives. The entire article selection and filtering process is illustrated in Figure 1.

**Figure 2:**
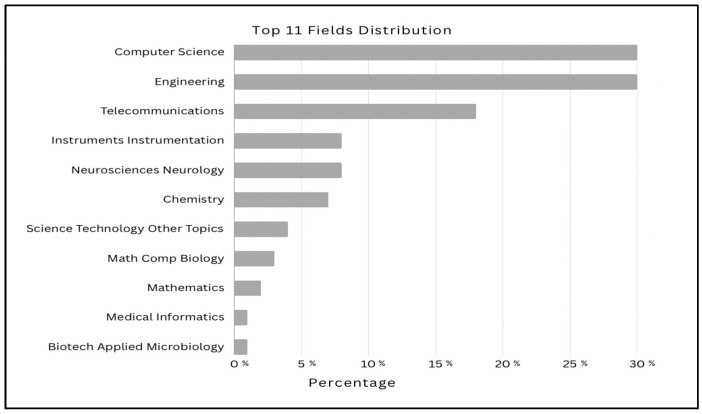
EEG signals in biometric authentication systems.

**Figure 3:**
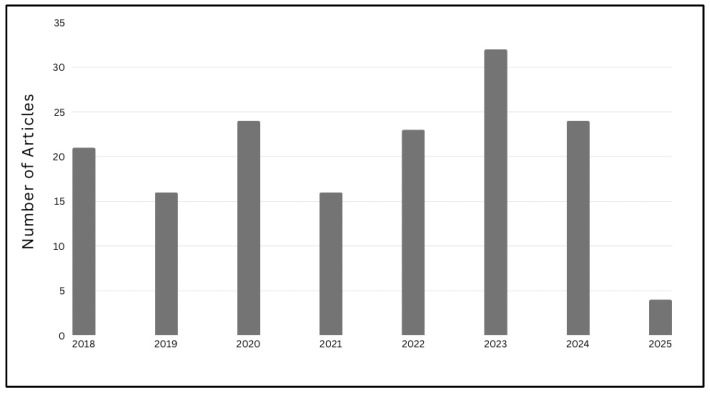
Annual Distribution of Published Articles (2018–2025).

The reviewed literature demonstrates a growing research focus on the utilization of EEG signals in biometric authentication systems. Figure 2 illustrates the thematic distribution of the top 11 categories within the selected studies retrieved from the WoS database. Moreover, Figure 3 presents the annual publication trends from 2018 to 2025, indicating a significant upward trajectory in EEG-based authentication research and highlighting the increasing academic and practical interest in leveraging brain signals for secure identity verification.

### 2.1. Machine Learning-Based Techniques

This section explores the utilization of classical machine learning algorithms in the context of EEG-based authentication. It reviews the preprocessing steps necessary to prepare EEG data for these algorithms, the feature extraction processes, and the performance metrics used to evaluate their efficacy.

TajDini et al. [8] proposed a novel approach for user authentication based on influencing human brain waves. Their study utilized a private dataset and employed fast Fourier Transform (FFT) for feature extraction. Classification was performed using a support vector machine (SVM). The model demonstrated an accuracy rate of 99.06%. Yousefi et al. [9] explored a novel approach to security authentication by predicting image memorability from users’ brain activity. Their study utilized a private dataset and employed power spectral density (PSD) for feature extraction. Classification was performed using SVM, achieving an accuracy rate of 88%.

Ortega-Rodríguez et al. [10] put forward a methodology for selecting and reducing the number of EEG sensors necessary to carry out the effective biometric identification of individuals. This system utilized both private and physioNet datasets. The method incorporates four techniques for extracting features: common spatial pattern (CSP), event-related desynchronization/synchronization (ERD/S), autoregression (AR), and FFT. By analyzing these features using SVM classifiers, they achieved a 99.99% accuracy rate.

Bak and Jeong [11] presented an Electroencephalography Motor Imagery (EEG-MI) methodology that utilized optimized feature extraction methods and classifiers to improve user-aware accuracy. They used the Big Data of 2 Classes MI dataset and PSD for feature extraction. The classification was performed using SVM, achieving an accuracy rate of 98.97%.

Alyasseri et al. [12] formulated the EEG channel selection problem as a binary optimization in which a binary version of the gray wolf optimizer (GWO) was used to find an optimal solution to such an NP-hard (nondeterministic polynomial-time hard) optimization problem. This approach employed the PhysioNet dataset and PSD for feature extraction. The classifier used was SVM, achieving a 94.13% accuracy rate. Baqer & Albermany [13] combined cryptography and biometrics to achieve authentication using a fuzzy vault scheme, employing the Graz N2a dataset and AR for feature extraction. The system used SVM for classification, achieving a 96.98% accuracy rate.

Arias-Carbacos et al. [14] investigated brain biometrics with consumer devices and introduced three novel techniques based on cognitive semantic processing. Their study employed the Brainwaves Authentication dataset and AR for feature extraction, with SVM as the classifier, achieving a 95.61% accuracy rate. Rahman et al. [15] proposed a novel multimodal biometric system combining EEG and keystroke dynamics. They used a private dataset and employed PSD for feature extraction. The classification was performed using random forest (RF), achieving a 99.6% accuracy rate. Cheng et al. [16] forwarded a hybrid BCI authentication approach that combined users’ EEG and eye movement data features simultaneously. Their study utilized a private dataset and employed PSD for feature extraction, achieving an accuracy of 88.35% using an RF classifier.

Sooriyaarachchi et al. [17] proposed a MusicID authentication solution for smart devices that used music-induced brainwave patterns as a behavioral biometric modality. They used a private dataset and employed statistical features including mean, maximum, minimum, and zero-crossing rate (ZCR) for feature extraction. Classification was performed using RF, achieving 99.46% for user identification and 97.31% for user verification accuracy rates. Yousefi and Kolivand [18] proposed a biometric authentication model that uses deep breathing patterns. They employed a private dataset and extracted features through discrete wavelet transform (DWT) and employed SVM and artificial neural network (ANN) classifiers, achieving average accuracies of 91% and 90%, respectively.

Shrivastava and Tcheslavski [19] demonstrated the efficacy of EEG as a biometric identifier. They employed a private dataset; PSD estimation and averaging were employed for feature extraction, followed by classification using ANN and Euclidean distance-based (ED) classifiers. The study reported a peak classification accuracy of 87.5% for both ANN and ED classifiers. Rathi et al. [20] utilized an EEG-based system for user authentication based on the P300 evoked by a customized visual stimuli paradigm. This approach used a private dataset, extracted features using the event-related potential (ERP) method, and achieved a high classification accuracy of 97% through quadratic discriminant analysis (QDA).

Wu et al. [6] proposed an EEG-based identity authentication method that is based on self-face or nonself-face rapid visual presentation. Their research used a private dataset and employed ERP for feature extraction. This was classified with hierarchical discriminant component analysis (HDCA), achieving a high accuracy rate of 91.31%.

Seha and Hatzinakos [21] proposed an approach for an EEG-based biometric recognition system using steady-state auditory evoked potentials (AEPs). They used a private dataset, and Gaussian filtering (GF) was employed for feature extraction. The study reported a high classification of 96.46% for the linear discriminant analysis (LDA) classifier.

Kaongeon et al. [22] proposed a two-factor authentication system that utilized the following knowledge factors: the knowledge of a client’s acquaintances as key and inherence factors and P300 ERP responses to visual stimuli as the medium. This study used a private dataset and employed ERP for feature extraction. The system used Fisher’s linear discriminant analysis (FLDA) for classification, achieving a 99% accuracy rate.

Behera et al. [23] proposed a new method to verify air signatures by analyzing finger movements and cerebral activities together with the help of sensors in next-generation consumer electronics (CE) devices. They used a private dataset and employed the hidden Markov model (HMM) in classification, achieving a 98.5% success rate. Alyasseri et al. [24] proposed a multiobjective binary version of the cuckoo search algorithm (MOBCS-KNN) to find optimal EEG channel selections for person identification. They used a PhysioNet dataset and employed k-nearest neighbors (k-NN) in classification, achieving a 93.86% success rate.

Rakshe et al. [25] proposed an EEG-based biometric authentication system incorporating emotion-induced stimuli to enhance the distinctiveness of neural signals. Among the classifiers evaluated, CatBoost achieved the highest accuracy of 91%. Oikonomou [26] presented an EEG-based biometric identification and verification framework combining Filter-Bank Common Spatial Patterns (FBCSPs) with Sparse Representation Classification (SRC). EEG signals were preprocessed and spatially filtered to extract subject-specific features. Classification via SRC achieved a CRR of 99.31% with 5 s SSVEP trials.

### 2.2. Deep Learning-Based Techniques

This section focuses on deep learning-based techniques, which have increasingly gained prominence due to their ability to automatically learn complex feature representations from raw EEG data. This section reviews various neural network architectures that have been proposed for EEG-based user authentication.

Buzzelli et al. [27] presented a unified deep learning framework for the recognition of user identity and the recognition of imagined actions based on EEG signals for application as a brain–computer interface. They used the PhysioNet dataset and employed AR as an extraction feature. Classification was performed using convolutional neural networks (CNNs), achieving an accuracy rate of 99.98%.

Debie et al. [28] proposed a novel EEG fusion method to examine the reliability and durability of EEG biometric markers across recording sessions. This study utilized the BCI Competition IV2a dataset and employed PSD for feature extraction. An accuracy rate of 98% was obtained after classification was performed using a CNN. Singh et al. [29] explored a new direction for selectively anonymizing a person’s brain signals resulting from a response to a stimulus. This research used the Brainwaves dataset and employed CNN for classification, attaining subject classification accuracy rates of 35% for binary classification and 38% for multi-class classification.

Xu et al. [30] proposed a novel deep learning framework called EEG-based Subject Matching Learning (ESML), which utilizes raw EEG signals as latent representations for EEG-based user identification and task classification. This study used Rapid Serial Visual Presentation (RSVP), Sternberg, and PhysioNet datasets, employing both AR and PSD as extraction features. The study reported a high classification of 99% for the CNN classifier.

Vadher et al. [31] presented an EEG-based multisubject and multitask biometric authentication system for military applications that addresses the challenges associated with multitask variation in EEG signals. The M3CV dataset was used in this study, and both AR and PSD were employed as extraction features. The system used a CNN for classification, achieving a 99.8% accuracy rate. Cui et al. [32] proposed an authentication approach for EEG databased on an attention mechanism and a triplet function. They used the PhysioNet dataset and employed AR as an extraction feature. Classification was performed using a CNN, achieving an accuracy rate of 99.97%.

Alsumari et al. [33] introduced a lightweight CNN model consisting of a small number of learnable parameters that enabled the training and evaluation of the CNN model on a small amount of available EEG data. The PhysioNet dataset was used, and both a CNN and long short-term memory (LSTM) were used as extraction features. Classification was performed using a CNN, achieving an EER rate of 0.187%. Monsy and Vinod [34] explored an EEG-based biometric identification model using a unique feature called frequency-weighted power (FWP). By employing both private datasets and PhysioNet, PSD for feature extraction, and a correlation-based classification approach, they achieved an unparalleled classification accuracy of 99.69%.

Kasim and Tosun [35] used photic stimuli with EEG data for the first time to identify both subjects with ADHD and healthy subjects. They utilized a private dataset and employed a CNN as a classifier, achieving an accuracy rate of 97.17%. Wu et al. [36] proposed a multitask EEG-based person authentication system by combining EEG signals with eye-blinking features, which allowed the system to achieve high precision and robustness. Their study utilized a private dataset and employed ERP for feature extraction, achieving an accuracy rate of 97.6% using a CNN classifier. Elshenaway and Guirguis [37] proposed an authentication approach for Internet of Things (IoT) devices that integrate EEG signals and hand gestures. This method utilizes a private dataset and a CNN for both feature extraction and classification. The approach achieved a significant accuracy level of 97.97%, indicating its effectiveness in correctly identifying authorized users.

Das et al. [38] proposed a spatiotemporal dense architecture for EEG-based person identification. Their research incorporated the PhysioNet dataset. The classification was performed using LSTM, achieving a 99.95% accuracy rate. Baler et al. [39] introduced an advanced EEG-based biometric identification system, termed DM-EEGID (Dense Multiscale EEG-based Identification), designed to achieve enhanced accuracy and robustness. The proposed system utilized the publicly available PhysioNet EEG dataset and integrated a hybrid architecture, wherein a CNN was employed for feature extraction, while a LSTM network functioned as the classifier. This hybrid model demonstrated remarkable performance, attaining an identification accuracy of 99.96%.

Tian et al. [40] combined different functional connectivity (FC) features within a graph convolutional neural network (GCN) framework, achieving high classification accuracy for unprocessed data. Their research utilized the PhysioNet dataset and employed RF for feature extraction. The classifier used was a GCN, achieving a 98.56% accuracy rate.

Xu et al. [41] proposed E-Key, a unified EEG-based framework for biometric authentication and fatigue detection in driving, utilizing a CNN–Attention model. The system achieved 98.5% accuracy in personal identification and outperformed CNN, CNN-LSTM, and Attention-based models, highlighting its promise for real-world in-vehicle applications.

Sharma et al. [42] proposed a motor imagery classification approach. EEG signals were converted into high-resolution time-frequency representations via the Superlet Transform (SLT) and classified using pretrained CNN models, achieving a classification accuracy of 96.4%.

### 2.3. SLR Findings and Discussion

This section provides a systematic analysis and critical discussion of recent studies related to authentication utilizing EEG data. It focuses on key aspects, such as authentication types, dataset characteristics, devices employed, and computational methodologies, including machine learning (ML) and deep learning (DL) approaches. The findings are contextualized within the broader domain of EEG-based authentication systems, highlighting the implications of these findings for future research and practical applications.

#### 2.3.1. Authentication Type

Various authentication types investigated in recent studies are classified into two primary categories: self-authentication (passive) and external authentication (active). Table 1 presents a summary of these authentication types.

Self-authentication relies exclusively on an individual’s unique EEG signals to verify their identity. This passive approach eliminates the need for users to perform specific actions during the authentication process, thus offering a seamless and unobtrusive experience. By leveraging the inherent distinctiveness of brainwave patterns, self-authentication provides a noninvasive mechanism for verifying identity without active user intervention.

Conversely, external authentication requires users to actively input secret combinations of characters—comprising letters, numbers, and occasionally special symbols—via EEG signals. These combinations serve as credentials to authenticate and validate a user’s identity. This method necessitates a more engaged interaction, with the user intentionally generating EEG patterns through cognitive or motor imagery tasks linked to the authentication credentials.

Our review of the literature revealed that 15 studies employed passive authentication systems, while 22 studies utilized active authentication systems. The preference for active systems in a majority of studies may stem from their potential to integrate additional layers of security by combining user-generated cognitive patterns with the inherent uniqueness of EEG signals. However, passive systems remain advantageous for applications in which minimal user engagement is preferred, such as continuous or seamless authentication scenarios. The insights from these studies underline the potential of EEG-based authentication methods to adapt to varying user needs and contexts, highlighting the trade-offs between security, usability, and user experience in passive versus active systems.

#### 2.3.2. Modality

Number of studies have demonstrated that combining modalities can significantly enhance authentication accuracy compared to single modalities. For example, Rahman et al. [15] developed a multimodal system that combines EEG with keystroke dynamics, achieving improved classification performance through machine learning methods. Cheng et al. [16] proposed a hybrid brain–computer interface model that integrates EEG and eye movement data to address shoulder-surfing threats. Behera et al. [23] introduced an authentication scheme for handheld devices that combines EEG features with 3D finger motion trajectories, enhancing resilience and usability. Additionally, Wu et al. [36] presented a method merging EEG signals with eye-blinking patterns for robust open-set person authentication. In our review, we found that the majority of the papers focused on a single modality, specifically EEG signals, while some utilized multiple modalities, as shown in Table 2.

#### 2.3.3. Dataset

The utilization of public datasets in EEG research has become a cornerstone for advancing methodologies and applications across various domains. Table 3 highlights several prominent EEG datasets that have been extensively reviewed for their contributions to diverse research areas. Among these, the Brainwave Authentication dataset [45] stands out as the only dataset specifically designed for biometric authentication, distinguishing it from others that primarily focus on motor imagery, RSVP paradigms, or general cognitive tasks.

The datasets used in the reviewed studies can be classified into two categories: public and self-collected. Public datasets are openly available resources shared to facilitate research, enabling broader analysis, benchmarking, and collaborative innovation. Conversely, self-collected datasets are generated independently for targeted research purposes, remain proprietary, and are typically inaccessible to the broader research community. Based on the review, 20 studies utilized public datasets, while 17 relied on self-collected datasets.

PhysioNet [46], one of the earliest public EEG datasets, was published on September 9, 2009. It includes 64-channel EEG recordings contributed by the developers of the BCI2000 instrumentation system. These recordings capture subjects performing motor and imagery tasks and have been instrumental in advancing the field of EEG-based research. Similarly, the BCI Competition IV-2a dataset [47], released in 2008, has been widely employed in motor imagery (MI) research. This dataset comprises 22-channel EEG recordings and serves as a benchmark for evaluating signal processing and classification techniques within the BCI community.

Another notable resource is the Big Data of 2-classes MI dataset [48], a substantial repository for MI research. It features 60 h of EEG recordings from 75 sessions involving 13 participants, providing a comprehensive foundation for developing and validating robust machine learning models tailored to MI tasks. Additionally, RSVP paradigms have contributed significantly to BCI research through datasets such as the RSVP dataset [49], published on 19 May 2017. This dataset comprises EEG recordings from 11 participants engaged in RSVP tasks at varying presentation rates, offering insights into the neural dynamics associated with rapid visual processing.

The M3CV dataset [50], a large-scale EEG database, features recordings from 106 subjects across multiple sessions and experimental paradigms. This extensive dataset supports the development of advanced ML algorithms by addressing intersubject and intrasubject variability, facilitating deeper insights into the generalizability of EEG signal processing techniques. Finally, the Brainwave Authentication dataset [45], comprising EEG recordings from 38 participants, was specifically designed to investigate the feasibility of brainwave-based biometric authentication. The dataset includes tasks that elicit distinct ERPs, such as P300 and N400, which are critical for distinguishing individual neural patterns. Its unique focus on authentication underscores its importance in advancing EEG-based biometric systems, setting it apart from the other reviewed datasets.

The DEAP dataset [51] is a multimodal dataset designed to support research in emotion recognition and affective computing. It comprises recordings from 32 participants who watched 40 one-minute-long music video excerpts while their EEG and peripheral physiological signals were simultaneously recorded. Each participant also provided self-assessments of their emotional states along valence, arousal, dominance, and liking dimensions.

The Speller dataset [52] contains EEG recordings from 35 subjects exposed to 40 distinct SSVEP stimulus frequencies (8–15.8 Hz). Signals were acquired using a 64-channel SynAmps2 system, with analysis focused on nine occipital and parietal–occipital channels. Each subject completed six blocks of 5 s trials, offering high-quality data for evaluating EEG-based biometric systems.

#### 2.3.4. Device

BCI headset devices represent an advanced technological frontier, enabling direct communication between the human brain and external systems by interpreting neural activity. These devices use EEG to capture and process brain signals, supporting diverse applications, such as BCI-based authentication. Table 4 summarizes the devices identified in the reviewed studies, highlighting their specifications and prevalence.

Among these, the Emotiv Epoc+, featured in 10 studies, emerged as the most utilized device. This portable EEG headset is equipped with 14 electrodes positioned at key sites (e.g., AF3, F7, F3, FC5, T7, P7, O1, O2, and P8) and employs saline-soaked felt pads for efficient and versatile EEG recording, making it a preferred choice for authentication studies. The B-Alert X10, used in eight studies, is a premier wireless EEG system optimized for mobile applications. It features nine high-quality EEG channels, with an additional optional channel for ECG, electromyography (EMG), or electrooculography (EOG). Sampling at 256 Hz, this device combines functionality with simplicity, making it suitable for a wide range of neurophysiological studies. Its widespread adoption across various peer-reviewed studies highlights its reliability and adaptability in capturing EEG data for diverse experimental settings.

#### 2.3.5. Computational Methods

Our review identifies a variety of computational methods and machine learning algorithms designed to enhance the accuracy and efficacy of BCI-based authentication systems. These methods are applied at different stages of the BCI system, specifically in signal processing, feature extraction, and classification. Table 5 presents a summary of the most commonly used methods at each stage, along with the corresponding number of studies and their references.

##### Signal Processing

Signal processing is a critical initial step in EEG data analysis. It involves various operations, such as averaging, filtering, noise removal, channel selection, and normalization. These techniques ensure that the data are of high quality and suitable for downstream computational tasks. Table 5 provides an overview of studies that employ these methods to preprocess EEG signals.

- Filtering was identified as the most frequently employed signal processing technique, appearing in 28 studies. This method is widely accepted for isolating relevant frequency bands associated with authentication. Filtering involves removing or emphasizing specific components of a signal based on their frequency or other characteristics, thereby enhancing the signal-to-noise ratio and focusing on the neural activity of interest [13].
- Noise Removal was utilized in seven studies to improve data quality and accuracy by eliminating irrelevant or unwanted signals. Techniques such filters, advanced algorithms, and other signal processing methods are employed to identify and remove noise, ensuring that the retained data are more representative of neural activity [9].
- Normalization adjusts data to a uniform scale, ensuring comparability across different signals or datasets. This technique facilitates direct comparisons by rescaling values within a predefined range, thus addressing the variability inherent in EEG data and enabling consistent analysis [8].
- Averaging is a statistical technique used to calculate the mean value of data points that is often employed to reduce the impact of random variations or noise. This approach is particularly useful in signal processing to smooth data, enhance the signal-to-noise ratio (SNR), and uncover underlying trends or patterns within the EEG signals [15].
- Channel Selection involves identifying and focusing on specific EEG channels or features that are most relevant to the authentication task. This method reduces the dimensionality of the data, improves computational efficiency, and enhances the focus of analysis on critical neural signals. By targeting specific channels, researchers can streamline data processing and improve the relevance of extracted features [6].

##### Feature Extraction

Feature extraction transforms raw EEG data into informative attributes, serving as the foundation for effective machine learning and analysis. The reviewed studies commonly utilized the following methods:

- Power spectral density (PSD) provides insights into brain activity by analyzing the power distribution of EEG signals across frequency bands [11].
- Event-related potentials (ERPs) are time-locked electrophysiological responses elicited by specific sensory, cognitive, or motor events, as recorded through EEG. These waveforms, characterized by distinct temporal and amplitude features, reflect neural mechanisms underlying processes such as perception, attention, memory, and decision-making.
- Renowned for their exceptional temporal resolution, ERPs offer profound insights into the dynamic functioning of the brain in response to external stimuli. Their application extends beyond cognitive neuroscience and clinical diagnostics to innovative fields, such as user authentication, where ERPs serve as potential neural biomarkers for identifying individual-specific cognitive patterns. This versatility underscores the significance of ERPs in advancing both the theoretical and applied domains of neuroscience [44].
- Autoregressive Models: These models analyze temporal relationships within EEG signals, offering valuable insights into signal dynamics [14].
- Other Methods: Additional techniques, such as discrete wavelet transform (DWT), Gaussian filtering (GF), random forest (RF), fully connected (FC) layers, and Filter-Bank Common Spatial Patterns (FBCSP) have been employed in previous studies. These methods offer diverse approaches to extracting significant features, each contributing unique strengths and limitations to EEG signal analysis.

The reviewed feature extraction methods exhibit varying strengths and weaknesses that influence their suitability for specific applications. For example, PSD is effective for identifying frequency domain characteristics, while ERP is more suited for capturing stimulus-evoked responses. Autoregressive models excel in analyzing temporal relationships, whereas advanced methods, such as DWT and GF, offer flexibility in addressing complex signal features. Selecting the appropriate feature extraction technique is critical for optimizing the performance of BCI-based systems, particularly in pain detection and authentication contexts.

##### Classification

Classification in BCI-based authentication involves applying DL and ML techniques to identify, interpret, and group EEG signals into predefined categories or classes. These approaches aim to recognize patterns in brainwave data that correspond to specific user identities. The classification models utilized in these studies range from conventional ML algorithms to advanced DL architectures, each offering unique capabilities for processing EEG signals.

Deep learning techniques have gained prominence due to their ability to model complex, nonlinear patterns in EEG data. Among these, CNNs are the most widely used architectures, excelling in spatial and temporal feature extraction from EEG signals. CNNs have been employed in numerous studies for their superior performance in identifying distinct neural patterns, as shown in Table 6.

In addition to CNNs, other DL methods have been explored. LSTMs are highly effective in modeling temporal dependencies, making them well suited for analyzing sequential EEG data. Additionally, GCNs leverage the inherent graph-like structure of EEG channel connectivity to model the spatial relationships between electrodes. Table 7 summarizes the studies employing these DL techniques.

Machine learning methods remain a cornerstone of BCI-based authentication, offering flexibility and interpretability in EEG signal classification. Prominent ML algorithms include SVMs, which are supervised learning algorithms that are widely recognized for their ability to efficiently separate data into distinct classes by finding an optimal decision boundary [12]. Table 8 lists the studies that utilized SVMs, underscoring their popularity in prior research.

In addition to SVMs, QDA models and classifies data by estimating the probability distribution of each user identity class, enabling the creation of nonlinear decision boundaries [44]. Similarly, LDA performs dimensionality reduction and classification by seeking linear combinations of features that maximize the separation between classes [21]. In addition to these models, a variety of other ML methods have been employed, such as KNN, RF, NN, HDCA, and FLDA. Table 9 summarizes the other ML classification methods.

The analysis of existing research underscores the relatively recent integration of BCI technology into authentication systems. This emerging field has witnessed the adoption of diverse methodologies for feature extraction and data classification, reflecting the evolving landscape of computational techniques in EEG-based authentication.

Among the feature extraction approaches, PSD and ERP methods are prominently utilized due to their efficacy in capturing the critical temporal and spectral characteristics of neural signals. These methods provide robust representations of EEG data, facilitating the accurate discrimination of user-specific brainwave patterns. In classification, CNNs and SVMs have emerged as the most commonly employed algorithms in the reviewed literature.

CNNs, with their ability to model complex spatial and temporal patterns in EEG signals, have demonstrated exceptional performance in extracting hierarchical features directly from raw data. Their adaptability to multichannel EEG inputs further enhances their suitability for authentication tasks. Conversely, SVMs, known for their robustness in handling high-dimensional datasets, offer a computationally efficient alternative to smaller datasets and scenarios requiring linear decision boundaries.

### 2.4. Challenges and Future Directions

BCI systems offer substantial potential for enabling direct communication between the human brain and external devices. Nevertheless, through an in-depth analysis presented in this literature review, we have identified key challenges within this field, which can be classified into three distinct categories: technological, user-related, and implementation. Table 10 provides a comprehensive overview of these challenges, alongside potential future directions for EEG-based systems, drawing on insights from previous studies.

Technological challenges, particularly sensor impedance and constraints in real-time processing capabilities, impede the efficacy of BCI systems, limiting their ability to achieve reliable data acquisition and optimal system usability. In the user domain, challenges such as insufficient user familiarity, inconsistencies in user ratings, and the complexity of the setup process further hinder the practical deployment of BCIs.

Moreover, the implementation of BCI systems is significantly affected by factors such as device obtrusiveness, limited information transfer rates, and high error rates. To propel the field forward, it is imperative to address these multifaceted challenges through ongoing advancements in sensor technologies, refinement of user interface design, and improved system integration.

Future research must focus on overcoming these barriers to enhance the performance, scalability, and broader applicability of BCI systems in diverse real-world environments.

## 3. BCI-Based Authentication System Design

**Figure 4:**
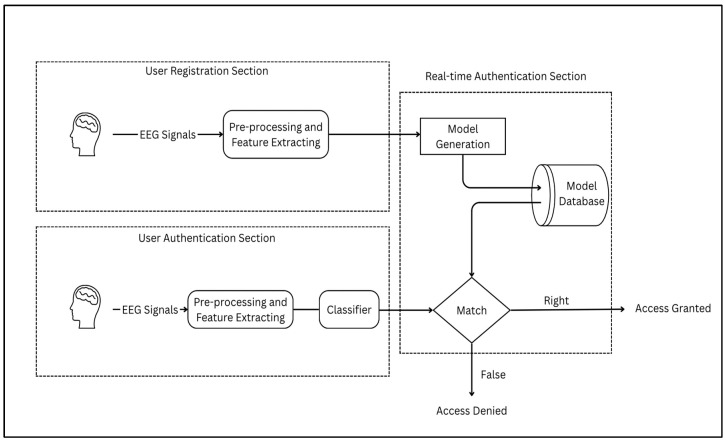
Flowchart describing a BCI-based system: registration and authentication phases.

The architecture of the BCI-based authentication system is structured into two phases: the registration phase and the authentication phase. The detailed flowchart outlining the sequential progression from registration to authentication within the BCI-based framework in Figure 4 provides a comprehensive overview of the workflow for both phases.

In the first step of the registration phase, brainwave signals from the user are captured via signal acquisition using EEG electrodes or channels, with data acquisition hardware recording electrical activity from the scalp. These raw EEG signals undergo data preprocessing, where techniques such as independent component analysis (ICA) and bandpass filtering are applied to reduce noise and artifacts, ensuring the accuracy of subsequent analyses.

Feature extraction follows, transforming preprocessed signals to extract essential information, guided by the literature to focus on ERPs and PSDs. Classification employs ML and DL methods, such as SVMs and CNNs, to categorize and recognize patterns, with the database model storing structured user data for referencing during authentication. The extraction of event-related neural features was guided by temporally structured physiological transitions, such as intermittent eye opening, which served as internal reference points for segmenting the EEG signal in the absence of conventional stimulus-locked events.

Moving to the authentication phase, the process is initiated via a frontend interface. The user engages in a similar EEG recording session under the same controlled conditions. The EEG signals are collected and transmitted to the backend, where they undergo preprocessing and feature extraction consistent with the registration pipeline. Matching evaluates the similarity between real-time EEG data and stored model data, generating an authentication score. Finally, the result presentation relays the output to the frontend, providing the authentication outcome to the user.

## 4. Experiment Design

The experimental study utilized the BCI authentication framework delineated in the preceding section in collaboration with participants who necessitate robust and reliable authentication solutions.

To operationalize this framework, the study implemented a rigorously structured EEG-based BCI system designed to verify user identity through semantic processing. Specifically, the study adopts an experimental paradigm grounded in semantic processing to evaluate the model’s ability to detect and authenticate individual identities. EEG data were recorded while participants engaged in a 20 min semantic mismatch task designed to elicit N400 responses. The participant cohort, drawn from diverse cultural backgrounds, enhances the generalizability of the findings. Data collection was conducted between 20 April and 24 April 2025, involving nine participants. Each underwent a structured three-phase protocol, including demographic data collection, experimental tasks, and a post-session user acceptance evaluation. The experimental procedure was informed by prior literature [45] on N400 elicitation through picture incongruency paradigms.

The following section provides a comprehensive account of the experimental protocol, encompassing ethical approvals, participant details, methodological procedures, and interface design. It begins by addressing ethical considerations, followed by an overview of participant demographics and rights. The experimental design and protocol are detailed, including participant instructions, EEG equipment, and data acquisition methods. Data analysis methodologies are then presented, along with the experimental results. Finally, the design of the user interface is discussed.

### 4.1. Ethics Statement

We obtained approval from the Institutional Review Board (IRB) under reference number 24-1295 at the College of Medicine Research Center (CMRC) at King Saud University (KSU) to ensure compliance with ethical standards regarding human subjects. Participants provided informed consent before participation, ensuring their voluntary involvement and the confidentiality of their data.

### 4.2. Participant Description

The study sample comprised nine female undergraduate students aged 18–22 enrolled at Imam Muhammad Ibn Saud Islamic University who voluntarily participated in the EEG experiment. A screening protocol was implemented to exclude individuals with a history of neurological or psychiatric disorders; all participants confirmed the absence of such conditions. Additionally, none of the participants had prior exposure to EEG or BCI technologies, thereby ensuring a standardized baseline of familiarity with the experimental setup.

The participants were recruited through email invitations, which included a comprehensive description of the experimental protocol and a link to a Google Form to indicate their consent to participate. To ensure the ethical conduct of the study, only individuals aged 18 years or older, capable of providing informed consent, were included in the sample. As part of the study, the participants were asked to express their willingness to permit the use of brainwave authentication as a primary method of authentication in the proposed model. These responses were systematically collected via the aforementioned Google Form.

### 4.3. Experimental Design and Protocol

The study aimed to evaluate the practicality of EEG authentication techniques. Conducted in a controlled laboratory environment, the experiment was designed to eliminate potential confounding factors, ensuring the reliability and validity of the collected data. The setup was carefully arranged to minimize external noise and maintain consistent testing conditions.

Participants were instructed to remain seated in a relaxed posture with minimal voluntary movement to reduce muscular and ocular artifacts. Throughout the 20 min recording, they performed a semantic mismatch task designed to elicit N400 responses, wherein each trial involved viewing a facial image followed by a word that either matched or mismatched the image. To preserve data integrity, participants maintained gaze fixation, cognitive neutrality, and refrained from swallowing or movements that could compromise signal quality.

### 4.4. EEG Equipment and Data Acquisition Software

**Figure 5:**
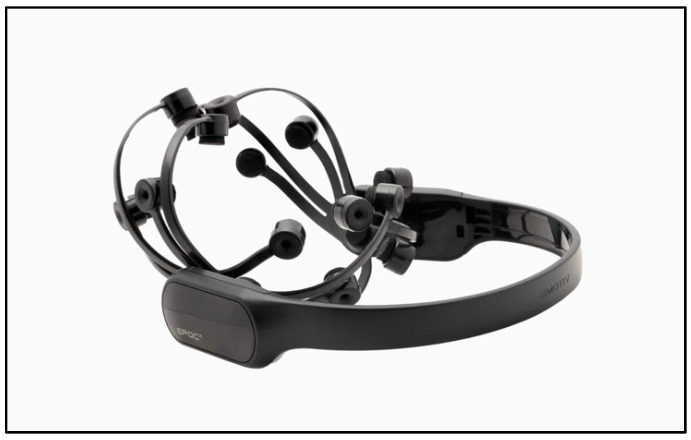
Emotiv EPOC X EEG device.

The experiment employed wireless EEG device (EPOC+, Emotiv, San Francisco, CA, USA) to capture the participants’ brain signals, as depicted in Figure 5. This advanced EEG system features 14 electrodes strategically positioned on the scalp to measure electrical activity in distinct regions of the brain. The Emotiv EPOC X samples EEG signals at a frequency of 128 Hz, which provides adequate temporal resolution for capturing event-related brain activity in cognitive tasks. The wireless connectivity and intuitive software of the Emotiv EPOC X render it particularly well-suited for academic and experimental research.

**Figure 6:**
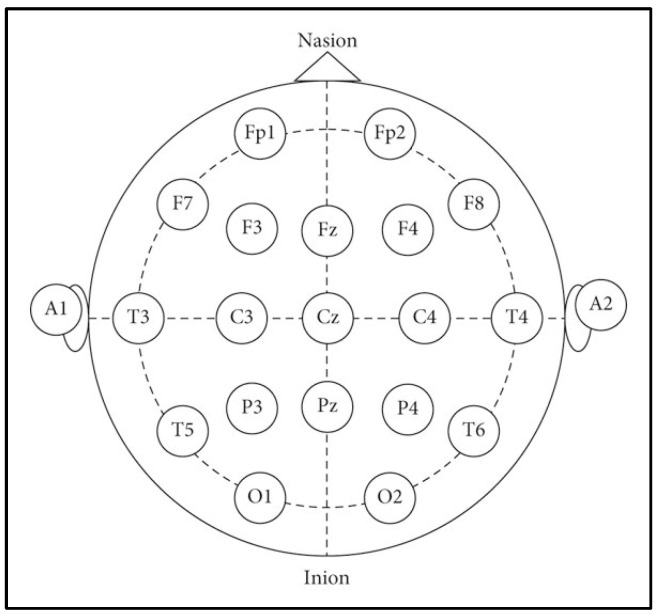
Electrode positions according to the international 10–20 system.

The channels corresponded to standard locations defined by the international 10–20 system, including AF3, F7, F3, FC5, T7, P7, O1, O2, P8, T8, FC6, F4, F8, and AF4. Figure 6 provides a schematic representation of the electrode positions following the 10–20 system. The data acquisition process was conducted in two distinct stages:

1. Training Stage: Each participant engaged in a one-minute session designed to train the computational model.
2. Testing Stage: Subsequently, a 10 s recording was captured to evaluate the model’s performance.

A one-minute rest period between recordings was provided to ensure data consistency and maintain electrode conductivity. This precaution minimized the risk of the conductive gel drying out and ensured optimal signal quality. The experimental setup was meticulously designed to reduce potential artifacts and interference, thereby improving the reliability of the recorded data.

**Figure 7:**
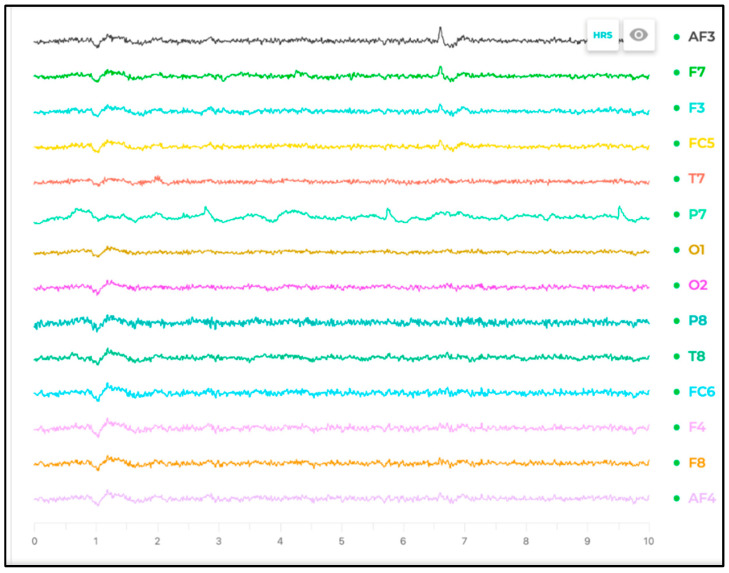
A participant’s EEG recording session using the Emotiv software.

Figure 7 illustrates a participant undergoing EEG signal recording using the Emotiv software interface, showcasing the user-friendly design and real-time feedback capabilities of the system.

### 4.5. Participants’ Response and Feedback

Upon concluding the experimental procedure, participants were cordially thanked, and each received a bar of chocolate as a token of appreciation. They were then directed to a Google Form to submit their responses to a user acceptance survey. The results from this survey indicate that all nine participants reported a successful experience with the session, reflecting a uniformly positive outcome across the cohort.

Participants found the authentication tasks readily comprehensible. Specifically, three participants assigned a usability rating of 4, two assigned a rating of 3, and four assigned a rating of 1, where 1 denotes ‘Strongly Agree’ and 5 denotes ‘Strongly Disagree’ in response to the statement ‘The authentication task was easy to complete. Regarding task-related confusion, five participants completed the tasks without any confusion (rating of 1), while the others indicated moderate confusion (rating of 3). Additionally, participants generally perceived the cognitive demands of the tasks as appropriate, with reported mental effort ratings predominantly ranging from 1 to 2, where 1 indicates ‘very low effort’ and 5 indicates ‘very high effort’.

Following a brief familiarization period, the majority of participants (7 out of 9) reported confidence in independently donning the headset. All participants affirmed that the headset was easy to put on and adjust. A substantial proportion (7 out of 9) also evaluated the device as highly comfortable for extended use, reflecting a positive overall experience with the hardware component.

Notably, none of the participants expressed any security-related apprehensions regarding the use of brainwave-based authentication, suggesting a high level of trust in the system’s integrity. When asked about their willingness to adopt brainwave authentication as their primary authentication method, all nine participants responded affirmatively, showing no hesitancy. Furthermore, every participant indicated that they would recommend the system to others, underscoring strong acceptance for the technology.

However, in some cases with extended EEG headset wear, several participants noted mild fatigue or discomfort due to prolonged headset use, with particular pressure on the forehead, and the need for occasional adjustment. These findings are consistent with prior studies which highlight the physical strain associated with extended EEG headset wear [56,57]. Moreover, participants unfamiliar with EEG systems reported initial difficulties in headset placement, underscoring the importance of intuitive, ergonomic hardware and clear onboarding instructions [58].

### 4.6. Data Analysis

We employed methodologies analogous to those detailed in Section 3 to construct predictive models based on the newly acquired dataset. The analytical framework adhered to the canonical stages of pattern recognition, encompassing preprocessing, feature extraction, and the application of classification algorithms. Notably, the preprocessing stage was refined to address noise artifacts arising from the data acquisition processes. The modifications to preprocessing are outlined below.

1. Data Preprocessing:
  - Data Cleaning: During preprocessing, nonessential columns, such as timestamp, original timestamp, and EEG counter, were excluded, and only the 14-channel EEG data were retained. Furthermore, rows containing general headset information, including serial numbers and firmware details, were removed. These steps streamlined the dataset, ensuring a focus on pertinent EEG channel data.
  - Data Consolidation: The data from all participants were merged into a single file, with an additional column introduced to uniquely identify each participant. This consolidation facilitated efficient aggregation and enhanced the dataset’s utility for subsequent analytical procedures.
  - Signal Filtering and Labeling: A bandpass filter (0.1–30 Hz) was applied to eliminate low-frequency drifts and high-frequency noise. A new categorical column, class, was introduced to assign unique subject labels, serving as the ground truth for model training and evaluation.

These refinements ensured the dataset’s integrity and optimized it for subsequent stages of analysis, thereby reinforcing the robustness of the prediction models.

2. Feature Extraction:

To capture both temporal and spectral characteristics of the EEG signals, two distinct types of features were extracted:

 
  - ERP Features: Continuous EEG data were segmented into one-second epochs, beginning from 100 ms prior to stimulus onset and extending to 900 ms poststimulus. This window was selected to encompass key ERP components while accounting for inter- and intra-subject variability in peak latency. The resulting event-locked features reflect transient cognitive responses with high temporal resolution.
  - PSD Features: Welch’s method was employed to compute PSD estimates for each channel within an epoch. The EEG signal was divided into overlapping windows, and FFT was applied with a segment size of 256 samples, yielding frequency-domain features indicative of underlying neural activity.

This combination of time-domain and frequency-domain representations enables the system to leverage both transient evoked responses and stable oscillatory patterns, offering a complementary framework for robust neural characterization within the context of biometric authentication. Numerous recent investigations have affirmed the efficacy of ERP- and PSD-based features in the context of EEG-based biometric systems. These features have exhibited strong discriminative capabilities and resilience across varying experimental paradigms [30,36]. Their integration within advanced classification frameworks, particularly deep learning architectures, has consistently yielded high performance in both user identification and authentication tasks.

3. Classification:

Following feature extraction, the dataset was compiled by aggregating non-overlapping segments derived from each participant’s EEG recordings. The initial 60 s session was exclusively utilized for training the classification model, while a separate 10 s session served for evaluation purposes. After preprocessing, the resulting dataset was partitioned into training and validation subsets using an 80/20 sample-wise split. This strategy ensured that validation was performed on unseen data, while maintaining balanced representation across all user classes.

**Figure 8:**
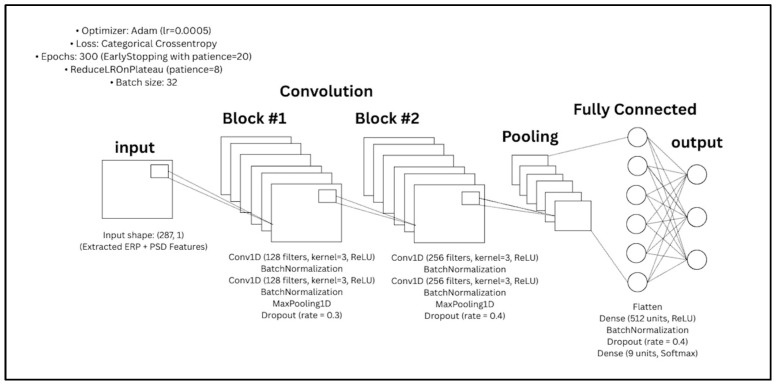
The CNN architecture.

The extracted features were fed into the CNN classifier, whose architecture is depicted in Figure 8. The CNN model is organized into sequential convolutional blocks followed by fully connected layers:

- First Convolutional Block: Two Conv1D layers with 128 filters and kernel size 3, each followed by batch normalization. MaxPooling1D and a dropout rate of 0.3 were applied to reduce spatial dimensions and prevent overfitting.
- Second Convolutional Block: Two Conv1D layers with 256 filters and kernel size 3, followed by batch normalization. A second MaxPooling1D layer and a higher dropout rate of 0.4 were applied.
- Dense Layers: The flattened output was passed through a fully connected layer with 512 units (ReLU activation), followed by batch normalization and 0.4 dropout. The final dense layer outputs probabilities over 9 user classes using softmax activation.

In total, the CNN model includes approximately 375,818 parameters, making it suitable for capturing intricate EEG signal representations. The network was compiled using the Adam optimizer and trained using categorical cross-entropy loss.

The model was trained over a maximum of 300 epochs with a batch size of 32. To mitigate overfitting and enhance generalization, an early stopping criterion was implemented with a patience threshold of 20 epochs, ensuring retention of the optimal model weights. A dynamic learning rate adjustment strategy was employed via ReduceLROnPlateau, configured to halve the learning rate upon stagnation in validation loss for 8 consecutive epochs. Furthermore, a model checkpointing mechanism was utilized to preserve the network parameters yielding the highest validation accuracy. No data augmentation procedures were introduced during training, thereby preserving the integrity of the original EEG signals throughout the learning process.

### 4.7. User Interface

As a critical component of this project, an advanced graphical user interface (GUI) was developed to operationalize the EEG-based authentication system, enabling real-time neural signal analysis for identity verification. The interface was meticulously designed to ensure smooth, efficient user interaction across both the registration and authentication phases.

**Figure 9:**
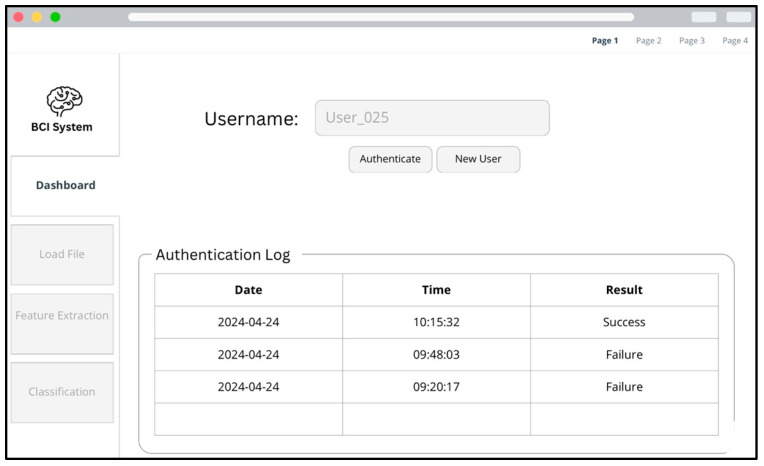
BCI system’s UI: dashboard view with authentication log and user controls.

The application was implemented in Python 3 using the Tkinter library and adopted a wizard-style design that guides users step by step through system setup and authentication procedures. As depicted in Figure 9, the first screen of the interface displays a dashboard containing an authentication log that summarizes previous attempts, with details such as date, time, and result. Clear controls are provided for registering a new user or initiating an authentication attempt.

**Figure 10:**
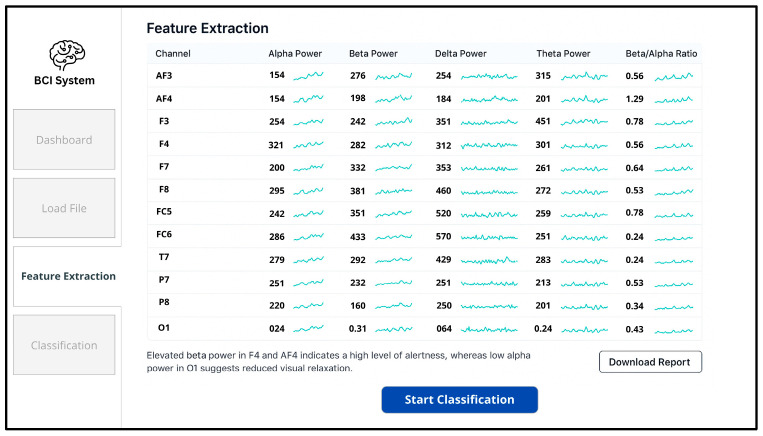
BCI system’s UI: feature extraction panel displaying spectral power metrics.

In the subsequent stage, shown in Figure 10, the interface allows the user to load EEG signal files (e.g., in edf format), confirms successful uploads, and visually presents the recorded neural signals in preparation for analysis.

**Figure 11:**
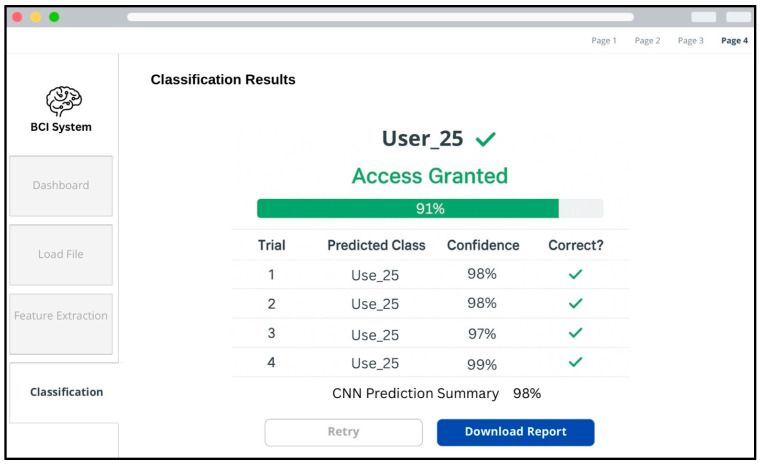
BCI system’s workflow and UI: classification results and access status.

Finally, the classification results are presented, as shown in Figure 11, where the system displays the predicted user identity, the confidence levels for each trial, and the overall CNN prediction summary. The interface visually confirms successful access authorization when classification outcomes meet the predefined acceptance criteria.

Overall, the user interface was purposefully crafted to optimize procedural flow, enhance usability, and ensure a seamless user experience, thereby supporting the effective deployment of the BCI authentication framework.

## 5. Result and Discussion

EEG recordings acquired over a one-minute interval were partitioned into training (80%) and testing (20%) subsets. The CNN model achieved 99% accuracy under this standard split of the dataset.

Across the various evaluation phases, the model consistently demonstrated strong performance. An accuracy of 98% was observed during a dedicated session under controlled conditions, while a brief, independent 10 s post-deployment segment yielded 97.7%, along with precision, recall, and F1 scores of 98%. Each evaluation scenario served to examine different aspects of the model’s behavior, from general classification performance to responsiveness under time-constrained, real-world conditions.

In the controlled setting, biometric error rates—FAR, FRR, and EER—were recorded at 2.2%, indicating a low incidence of classification errors. Evaluation metrics were derived in accordance with established biometric authentication standards. The FAR quantifies the proportion of unauthorized access attempts that were erroneously accepted as legitimate. Conversely, the FRR measures the proportion of legitimate user attempts that were incorrectly denied access. The EER denotes the threshold at which FAR and FRR intersect, offering a unified indicator of the system’s overall classification reliability.

In addition to CNN-based evaluations, traditional machine learning classifiers were also implemented for comparative analysis. The following classifiers were assessed: LR, RF, SVM, NB, DT, KNN, and GB. Table 11 summarizes each model’s performance metrics, including accuracy, precision, and recall. Each traditional classifier was configured using standard hyperparameters grounded in prior EEG-based classification studies. LR was implemented with L2 regularization (C = 1.0, solver = lbfgs), offering a baseline linear model known for its generalization capability and interpretability. SVM, configured with a radial basis function (RBF) kernel (C = 1.0, gamma = ‘scale’), was selected for its capacity to handle high-dimensional, nonlinear decision boundaries effectively. NB was included due to its probabilistic nature, computational efficiency, and demonstrated utility in EEG classification where feature independence assumptions may approximately hold. DT was employed for its inherent interpretability and capacity to capture nonlinear relationships without the need for extensive preprocessing.

KNN was configured with k = 5, uniform weighting, and Euclidean distance; it was chosen for its simplicity and non-parametric nature, making it particularly suitable for scenarios involving small-to-medium scale EEG datasets.

Compared to traditional classifiers, CNNs offer a more robust mechanism for capturing both spatial and temporal dependencies inherent in multichannel EEG signals. Their capacity for automatic feature learning and adaptability to raw, high-dimensional inputs enables superior generalization, making them especially advantageous for EEG-based biometric authentication tasks.

As shown in the table, the RF and GB classifiers achieved notably high performance, with accuracy rates of 95% and 94%, respectively, closely approaching the results obtained by the CNN model. Conversely, classifiers such as SVM and KNN exhibited comparatively lower performance, reflecting their limited capacity to model the complex patterns inherent in EEG data. These comparative results further validate the superiority of deep learning approaches. These comparative outcomes highlight the inherent trade-offs among the classifiers in terms of accuracy, computational complexity, and interpretability. CNN outperformed all traditional models, albeit with higher computational demands. Tree-based models like RF and GB offered a favorable balance between performance and efficiency, while simpler models such as NB and LR remained limited in capturing nonlinear EEG patterns but provided fast and interpretable results.

To strengthen the contextual analysis of our findings, we compared the performance of our proposed CNN-based system with recent contributions in the field that similarly employed convolutional neural networks for EEG-based authentication. These studies—reviewed earlier in the literature—offer a relevant benchmark for evaluating the efficacy of different feature extraction strategies and model designs.

Table 12 presents a consolidated overview of these studies, detailing the feature extraction strategies employed and the corresponding classification accuracies. As illustrated, the proposed system achieved an accuracy of 99% by integrating both ERP and PSD features, which aligns closely with the performance reported in [30], where a combination of AR and PSD was used. In contrast, studies that relied on single-domain features—such as PSD [37] or AR [32]—exhibited lower accuracy, ranging between 90% and 99%, underscoring the potential benefit of multi-domain feature integration. The highest performance was reported in [34], where raw EEG signals were processed using a deep CNN architecture to attain 99.96% accuracy; however, such approaches may come with increased computational complexity and reduced interpretability. Collectively, these results reaffirm the strength of our feature design and model structure in achieving competitive, state-of-the-art performance with balanced efficiency and practical applicability.

However, the current work is subject to certain limitations. The experimental setup involved a small and demographically homogeneous participant pool, which inherently limits the generalizability of the results to broader populations. Moreover, the absence of longitudinal experimentation precludes a robust assessment of the system’s temporal stability—an important factor given the variability of EEG signals in response to cognitive fatigue, emotional fluctuations, and environmental conditions. This observation aligns with previous literature emphasizing the significance of user-centered design in reducing cognitive barriers and enhancing accessibility

Furthermore, users recommended enhancements to the interface, such as improved visual clarity and simplified navigation. These suggestions reinforce the need for iterative refinements to both hardware and software components in order to maximize usability, comfort, and long-term acceptance of EEG-based authentication platforms [58].

Future research should address these constraints by incorporating substantially larger and more heterogeneous participant cohorts—encompassing diversity in gender, physiological attributes, and cognitive backgrounds—alongside repeated measurements across multiple sessions. Such advancements are crucial for validating the long-term reliability, adaptability, and real-world applicability of EEG-based authentication systems. Additionally, future work will explore hybrid approaches integrating EEG with complementary biometric modalities, expand publicly available datasets to enhance training scalability, and investigate advanced machine learning models to further optimize classification performance.

In addition, real-world implementation of EEG-based authentication systems necessitates careful consideration of operational constraints. These include the susceptibility of EEG signals to environmental noise and physiological artifacts, the computational demands associated with real-time signal processing, and the logistical challenges of scaling such systems for broader adoption—including user training, device calibration, and long-term maintenance. Future research should prioritize addressing these practical limitations to ensure the system’s viability beyond controlled experimental settings.

## 6. Conclusions

This paper presents a systematic review and an innovative framework for exploring EEG-based authentication systems. We have thoroughly examined existing literature and conducted an experimental evaluation to assess the feasibility, limitations, and scalability of these systems in practical applications. Our findings indicate that the CNN model achieved impressive accuracy rates, surpassing 99%, while other classifiers like Random Forest and Gradient Boosting also performed well, demonstrating the potential of EEG data in enhancing security measures.

Overall, this research emphasizes the significant potential of EEG-based authentication as a robust alternative to traditional methods, paving the way for more secure and user-friendly identity verification technologies.

## Figures and Tables

**Table 1:** Authentication type across related studies.

Authentication Type	Number of Studies	References
Passive	15	2024: [25]
		2023: [8,18,27,30,33,41]
		2022: [12,24,32,39,40]
		2020: [34,43]
		2019: [38]
Active	22	2025: [26,42]
		2024: [31]
		2023: [10,11,14,16,29]
		2022: [13,28]
		2021: [15,17,20,23,35,37]
		2020: [21,22,44]
		2018: [6,19,36]

**Table 2:** Multimodal EEG authentication studies.

Ref.	Modalities Combined	Signal Processing	Feature Extraction	Classification Method	Accuracy
[15]	EEG + Keystroke Dynamics	Filtering	PSD	RF	99.6%
[16]	EEG + Eye Movement	Filtering	DWT	RF	88.35%
[23]	EEG + 3D Finger Motion	Filtering	N/A	Hmm	98.5%
[36]	EEG + Eye Blinking Patterns	Normalization + Averaging	ERP	CNN	97.6%

**Table 3:** Several prominent EEG datasets based on our review.

**Dataset**		**No. of** **Subjects**	**References**
**Public dataset**	PhysioNet	9	[10,12,24,27,30,32,33,34,38,39,40]
	BCI competition IV-2a	54	[13,42]
	Big Data of 2-classes MI	9	[11]
	RSVP	11	[30]
	M3CV	106	[31]
	DEAP	32	[25]
	Speller	35	[26]
	Brainwave Authentication	38	[14,29]
**Private dataset**			[6,8,9,15,16,17,18,19,20,21,22,23,35,36,37,41,44]

**Table 4:** List of devices used in previous research.

Device	References
Emotiv EPOC+	[6,9,10,14,15,18,19,23,29,43]
B-Alert X10	[12,24,27,32,33,38,39,40]
BrainAmp	[11,34]
g.USBamp	[30,36]
Muse headset	[35]
NeuroSky MindWave	[26,37,44]
Waveguard	[44]
Cognionics HD-72	[41]
BioSemi ActiveTwo system	[25]

**Table 5:** Computational methods in related research.

Computational Methods		No. of of Studies	References
Signal Processing	Averaging	3	[21,25,36]
	Filtering	28	[6,8,9,11,13,14,15,16,18,19,20,21,22,23,25,26,28,29,30,33,34,35,36,38,41]
	Noise removal	11	[8,9,14,16,21,25,26,31,37,41,42]
	Channel selection	4	[25,26,34,42]
	Normalization	6	[8,9,20,36,39]
Feature Extraction	PSD	8	[9,11,12,15,19,28,30,37]
	ERP–P300	4	[6,20,22,44]
	ERP–N250	1	[36]
	AR	5	[10,13,14,30,32]
	Others (DWT, GF, RF, FC, SLT, and FBCSP)	7	[16,18,21,26,38,40,42]
Classification	CNN	13	[27,28,29,30,31,32,33,34,35,36,37,41,42]
	LSTM	2	[38,39]
	SVM	7	[8,9,10,11,12,13,14]
	NN	2	[18,19]
	RF	2	[15,17]
	QDA	2	[20,44]
	Others (GCN, HDC, LDA, FLDA, HMM, CatBoost, SRC, and KNN)	9	[6,16,21,22,23,24,25,26,40]

**Table 6:** CNN classification models in related research.

Ref.	Feature Extraction	Dataset	No. of Subjects	Evaluation			
				Accuracy	Precision	Recall	F1
[37]	PSD	Private dataset	30	97.97%	97.98%	97.92%	97.95%
[36]	ERP		40	97.6%	N/A		
[35]	N/A		16	97.17%	96.37%	96.59%	96.43%
[34]	CNN		16	99.96%	N/A		
[33]	CNN and LSTM	PhysioNet	109	EER = 0.187%	N/A		
[32]	AR			99.97%	N/A		
[27]	AR			99.98%	95.9%	91.8%	93.8%
[30]	AR and PSD			99%	99.5%	98.7%	99.1%
[28]	PSD	BCI Competition IV-2a	54	95%	N/A		
[29]	N/A	Brainwave Authentication	37	38%	N/A		
[31]	N/A	M3CV	95	99.86%	N/A		
[41]	N/A	Cognionics HD-72	31	98.5%	N/A		
[42]	SLT	BCI Competition IV-2a	54	96.4%	N/A		

**Table 7:** DL-based classification techniques.

Ref.	Feature Extraction	Classification	Dataset	No. of Subjects	Evaluation			
					Accuracy	Precision	Recall	F1
[43]	SMAT	Unique respective algorithm model for each user	Private dataset	24	N/A			
[38]	FC	LSTM	PhysioNet	109	99.95%	N/A		
[40]	RF	GCN			98.56%	N/A		
[39]	CNN	LSTM			99.96%	99.94%	99.94%	99.94%

**Table 8:** SVM classification models in related research.

Ref.	Feature Extraction	Dataset	No. of Subjects	Evaluation			
				Accuracy	Precision	Recall	F1
[8]	FTT	Private dataset	50	99.06%	N/A		
[9]	PSD		20	88%	N/A		
[10]	CSP, ERD, AR, FTT	Private dataset PhysioNet	13 109	99.9%	99.2%	99.2%	99.1%
[11]	PSD	Big Data of 2 classes MI	54	98.97%	99.75%	99.69%	99.68%
[12]	PSD	PhysioNet	109	94.13%	93.21%	92.91%	92.85%
[13]	AR	Graz IV2a	9	96.98%	N/A		
[14]	AR	Brainwaves Authentication	39	95.61%	95.50%	95.36%	95.38%

**Table 9:** ML-based classification techniques.

Other classification								
Ref.	Feature Extraction Method	Classification	Evaluation					
			Dataset	No. of Subjects	Accuracy	Precision	Recall	F1
[18]	DWT	NN	Private dataset	50	91%	95%	89%	85%
[19]	PSD	NN		8	87.5%	N/A		
[15]	PSD	RF		10	99.9%	N/A		
[17]	AI and PLV	RF		20	95%	87.1%	86.4%	85.5%
[20]	ERP	QDA		10	97%	96.7%	97.2%	96.9%
[44]	ERP	QDA		10	96.78%	N/A		
[6]	ERP	HDCA		45	91.31%	N/A		
[21]	GF	LDA		30	96.46%	N/A		
[22]	ERP	FLDA		10	99%	N/A		
[16]	DWT	RF		20	88.35%	N/A		
[23]	N/A	HMM		20	98.5%	N/A		
[24]	N/A	KNN	PhysioNet	109	93.86%	91.46%	92%	91.5%
[25]	N/A	CatBoost	BioSemi ActiveTwo system	32	91%	92%	91%	91%
[26]	FBCSP	SRC	Speller	35	99.31%	N/A		

**Table 10:** Challenges and future directions in EEG-based systems.

Type	Ref.	Challenges	Potential Future Directions
**User-Related**	[13]	Challenges within the user domain, including a lack of familiarity with the system, inconsistent user ratings, and the labor-intensive nature of setup procedures	Future research should focus on enhancing user training protocols, establishing standardized rating methodologies, and streamlining the setup process to improve user experience and reduce inconsistencies.
	[17]	Variability in individual responses to music-induced brainwave patterns introduces inconsistencies, challenging the reliability of EEG-based systems	Future research should explore methods to account for individual variability, perhaps through personalized calibration or adaptive models that accommodate diverse responses to auditory stimuli.
**Technological**	[14]	Achieving reliable classification and feature extraction is hindered by the low SNR of EEG signals and the intrusion of artifacts from various sources, such as hair, fatty tissue, and measurement equipment	Advancements in signal processing techniques are necessary, with a focus on artifact removal and noise reduction to enhance the quality and reliability of EEG signal extraction.
	[30]	The adoption of dry-electrode EEG headsets in nonlaboratory environments results in reduced signal quality, which undermines long-term reliability	Future developments should aim at optimizing dry-electrode technology to improve signal fidelity in real-world conditions, with the potential for more reliable and practical applications.
	[53]	EEG signals are highly susceptible to environmental noise and motion artifacts in real-world settings, leading to signal degradation and reduced authentication accuracy.	Future studies should validate EEG systems in ecologically valid environments and develop robust artifact-removal algorithms tailored for dynamic, noisy contexts.
	[54]	Real-time deployment of EEG-based authentication systems is constrained by high computational demands of deep learning models.	Future studies should focus on optimizing lightweight models and efficient processing pipelines suitable for real-time applications and resource-constrained devices.
**Implementation**	[32]	The study’s small and homogeneous sample size, with a narrow demographic range, limits the generalizability of its findings, particularly in relation to demographic and physiological variability	Future studies should prioritize larger, more diverse participant pools to enhance the generalizability and applicability of EEG-based biometric systems across varied demographic and physiological profiles.
	[19]	The small sample size in this study limits the robustness and applicability of the findings, with the risk of overfitting and poor generalization to broader populations	Research should focus on increasing sample sizes and ensuring more diverse demographic representation to strengthen the robustness and generalizability of EEG-based models across broader populations.
	[15]	Scaling EEG authentication systems to large user populations is hindered by the need for individual calibration, user training, and ongoing maintenance	Future work should explore user-independent or semi-supervised models, reduce calibration time, and automate system updates to support scalable deployment.
	[55]	Integrating EEG technologies into real-world 5G-IoT infrastructures presents significant challenges due to device incompatibility and protocol heterogeneity	Future work should prioritize the development of standardized APIs and integration frameworks to ensure interoperability and secure, seamless system coordination.

**Table 11:** Performance metrics of traditional machine learning models.

Classifier	Accuracy	Precision	Recall	F1
CNN	99%	99%	99%	99%
GB	93%	93%	93%	93%
KNN	55%	63%	54%	56%
DT	81%	82%	81%	81%
NB	63%	68%	63%	60%
SVM	48%	64%	48%	49%
RF	94%	94%	94%	94%
LR	60%	62%	60%	60%

**Table 12:** Comparative CNN-Based Results.

Ref.	Feature Extraction Method	Classification	Accuracy
[37]	PSD	CNN	97.97%
[36]	ERP		97.6%
[34]	CNN		99.96%
[32]	AR		99.97%
[30]	AR and PSD		99%
[28]	PSD		95%
Our work	ERP and PSD		99%

## Data Availability

Dataset used in this study is available upon request.
